# Lrba participates in the differentiation of IgA+ B lymphocytes through TGFβR signaling

**DOI:** 10.3389/fimmu.2024.1386260

**Published:** 2024-06-21

**Authors:** José Mizael Flores-Hermenegildo, Felipe de Jesús Hernández-Cázares, Daniela Pérez-Pérez, Héctor Romero-Ramírez, Juan Carlos Rodríguez-Alba, Paula Licona-Limon, Manfred W. Kilimann, Leopoldo Santos-Argumedo, Gabriela López-Herrera

**Affiliations:** ^1^ Departamento de Biomedicina, Centro de Investigación y de Estudios Avanzados del Instituto Politécnico Nacional (CINVESTAV), Ciudad de México, Mexico; ^2^ Laboratorio de Inmunodeficiencias, Instituto Nacional de Pediatría (INP), Ciudad de México, Mexico; ^3^ Programa de Doctorado en Ciencias Biológicas, Universidad Nacional Autónoma de México (UNAM), Ciudad de México, Mexico; ^4^ Unidad de Neuroinmunología y Neurooncología, Instituto Nacional de Neurología y Neurocirugia (NINN), Ciudad de México, Mexico; ^5^ Facultad de Medicina y Cirugía, Universidad Autónoma Benito Juárez de Oaxaca (UABJO), Ciudad de Oaxaca, Mexico; ^6^ Departamento de Biología Celular y del Desarrollo, Instituto de Fisiología Celular, Universidad Nacional Autónoma de México, Ciudad de México, Mexico; ^7^ Department of Molecular Neurobiology, Max-Planck-Institute for Multidisciplinary Sciences, Göttingen, Germany

**Keywords:** Lrba, TGFβR, B cells, SMAD2 phosphorylation, IgA

## Abstract

**Introduction:**

Lrba is a cytoplasmic protein involved in vesicular trafficking. *Lrba*-deficient (*Lrba-/-*) mice exhibit substantially higher levels of IgA in both serum and feces than wild-type (WT) mice. Transforming growth factor β1 (TGFβ1) and its receptors (TGFβR I and II) is essential for differentiating IgA+ B cells. Furthermore, increased IgA production suggests a potential connection between Lrba and the TGFβR signaling pathway in IgA production. However, the specific function of Lrba in B cell biology remains unknown.

**Aim:**

Given the increased IgA levels in *Lrba*-/- mice, the goal in this work was to explore the lymph organs where the switch to IgA occurs, and if TGFβR function is affected.

**Methods:**

Non-immunized *Lrba-/-* mice were compared with *Lrba+/+* mice. IgA levels in the serum and feces, as well as during peripheral B cell development, were determined. IgA+ B cells and plasma cells were assessed in the small intestine and secondary lymphoid organs, such as the spleen, mesenteric lymph nodes, and Peyer’s patches. The TGFβR signaling pathway was evaluated by determining the expression of TGFβR on B cells. Additionally, SMAD2 phosphorylation was measured under basal conditions and in response to recombinant TGFβ. Finally, confocal microscopy was performed to investigate a possible interaction between Lrba and TGFβR in B cells.

**Results:**

*Lrba-/-* mice exhibited significantly higher levels of circulating IgA, IgA+ B, and plasma cells than in peripheral lymphoid organs those in WT mice. TGFβR expression on the membrane of B cells was similar in both *Lrba-/-* and *Lrba+/+* mice. However, intracellular TGFβR expression was reduced in *Lrba-/-* mice. SMAD2 phosphorylation showed increased levels under basal conditions; stimulation with recombinant TGFβ elicited a poorer response than in that in *Lrba+/+* B cells. Finally, we found that Lrba colocalizes with TGFβR in B cells.

**Conclusion:**

Lrba is essential in controlling TGFβR signaling, subsequently regulating SMAD2 phosphorylation on B cells. This mechanism may explain the increased differentiation of IgA+ B cells and production of IgA-producing plasma cells.

## 1 Introduction

Lipopolysaccharide (LPS)-responsive beige-like anchor (LRBA) is a cytoplasmic protein belonging to the beige and Chediak-Higashi syndrome (BEACH) family of proteins associated with vesicular trafficking processes (1). This deficiency affects regulatory T cells (Treg) function. The human phenotype was first described in 2012; biallelic mutations in LRBA are associated with immunodeficiency characterized by autoimmunity, recurrent infections, defects in B cell activation, decreased class-switched memory B cells, and low IgG and IgA levels (2, 3). LRBA is associated with the endomembrane system, including the endoplasmic reticulum, endocytic vesicles, lysosomes, and Golgi apparatus, suggesting its participation in vesicular trafficking (4). Additionally, LRBA has been shown to interact with cytotoxic T-lymphocyte-associated protein 4 (CTLA4) in endosomes; such interactions are essential for recycling this protein, and therefore, LRBA-deficient Treg cells show reduced CTLA4 expression and increased degradation (5).

The LRBA protein is 90% homologous to the murine sequence. Lrba is ubiquitously expressed and is induced two- to four-fold in immune cells after stimulation with LPS (4). *Lrba-/-* murine deficiency also exhibited reduced CTLA4 expression in Tregs. In B cells, *Lrba-/-* mice showed increased IgA levels in the serum and small intestine (6).

The class switch recombination to IgA can be carried out by two pathways: T-cell dependent or independent. In the T cell-dependent pathway, activated T cells interact with B cells through the CD40 ligand, (CD40L), and CD40 receptors, inducing the expression of activation-induced cytidine deaminase (AID), leading to class switch recombination (7). Cytokine secretion by activated T-cells guides the switch to specific immunoglobulins. In the case of IgA induction, B lymphocytes require transforming growth factor β1 (TGFβ1), a cytokine secreted by various cell types, including several subsets of CD4+ T cells (8).

In the case of T-independent IgA induction, antigens interact with B cells and multiple innate immune cells through either the B-cell receptor (BCR) or Toll-like receptors (9, 10). Additionally, dendritic cells release B-cell-activating factor (BAFF; also known as BLyS) and proliferation-inducing ligand (APRIL). APRIL induces AID expression, while TGFβ1 is required to direct the isotype switch to IgA (11–14).

TGFβ1 is a pleiotropic cytokine belonging to the TGFβ superfamily. It is derived from the proteolytic cleavage of latency-associated peptide (LAP) with subsequent dimerization (15). TGFβ receptor (TGFβR), in B cells is composed of two of each TGFβRII and TGFβRI subunit, both with kinase activity. Upon TGFβ1 interaction with TGFβRII, phosphorylation of TGFβRI occurs, leading to its activation and subsequent phosphorylation of receptor-regulated SMAD proteins, including SMAD2 and SMAD3. The phosphorylation of these proteins causes their association with SMAD4 in the cytoplasm. These complexes are then translocated to the nucleus where they bind to SMAD-binding elements. One target of these SMAD heterodimers is the constant α heavy chain (IgHα) (7, 8).

The relevance of TGFβR in the class switch to IgA has been demonstrated in conditional murine-deficient models. Specifically, B cells deficient in TGFβRII showed reduced IgA+ B cells in both spleen and Peyer patches (PP), along with low levels of IgA in the serum (16).

Given the increased IgA levels in *Lrba-/-* mice, we aim to explore the lymph organs where the switch to IgA occurs, and if TGFβR function is affected. Here, we explored the presence of IgA+ B and plasma cells in secondary lymphoid organs, as well as TGFβR expression and SMAD2 phosphorylation under both basal and activation conditions; finally, the possible interaction between Lrba and TGFβR was explored by co-localization experiments, the data obtained suggest a direct role for Lrba in controlling TGFβR signaling.

## 2 Materials and methods

### 2.1 Experimental animals

*Lrba*-deficient mice were (6, 17) engineered at Taconic Artemis (Köln, Germany) (official allele designation: Lrbatm1.1Kili MGI: 5796558; laboratory designation: Lrba2) by constitutive deletion of the coding exon 3. This deletion introduced a frameshift mutation that was predicted to produce a truncated protein that was prone to degradation. Consequently, a shift in the reading frame occurred after amino acid 149 (5% of the coding sequence), rendering the expression of Lrba undetectable by Western Blot analysis.

The mice were bred and maintained in a C57BL/6 N gene pool under pathogen-free conditions at the animal facilities of the Research Centre and Advanced Studies (CINVESTAV, Mexico City, Mexico). Genotyping was performed using PCR amplification of genomic DNA extracted from splenocytes or tail samples using the following primer combinations: a sense primer for exon 3 (Ex3F: 5′-GAAAGTTGACAGTATGATTGCAGG-3′) paired with a wild-type-specific reverse primer in exon 4 (Ex4R: 5′-CATTGTCCTTTATCTCCTTGAA-3′), or a combination of Ex3F and a reverse primer for intron 4 (Int4R: 5′-CTAAGGAGGATGGCTCTAACC-3’).

This study was reviewed and approved by the CINVESTAV Ethics Committee and all the mice were maintained according to the Institutional Animal Guidelines for Animal Care and Experimentation (protocol number: 145–15, UPEAL-CINVESTAV-IPN).

### 2.2 Immunoglobulin levels quantification by ELISA

Ninety-six-well plates were coated with 5 ng/well of the capture antibody, anti-mouse IgG, IgA, or anti-IgM (SouthernBiotech, Birmingham, AL, USA) and incubated overnight at 4°C. The plates were blocked with 1% milk in 1X PBS containing 0.05% Tween for 2 h. Samples, including sera or fecal supernatants diluted at a 1:100 ratio in PBS, were added to the wells and incubated for 1 h at 37°C. Subsequently, biotinylated antibodies specific for either IgM (Southern Biotech, 102008), IgG (Southern Biotech, 103005), or IgA (Southern Biotech, 104008) were diluted at 1:1000 and added to the plates, followed by incubation for 1 h at 37°C. Finally, streptavidin was added and coupled with horseradish peroxidase (HRP; Abcam, Cambridge, UK, ab7403) diluted at 1:5000. Tetramethylbenzidine was added, and the reaction was stopped with 0.2 M phosphoric. The absorbance was measured at 450 nm using an ELISA reader (Microplate, Sunrise™).

### 2.3 Splenic B cell subpopulations and B1 immunophenotyping

Splenocytes were stained with anti-CD19 BV421, anti-CD23 PerCPCy5.5, anti-CD21 PE, and anti-IgM PECy7 (all from Biolegend, San Diego, CA, USA) to detect Transitional 1, Transitional 2, Follicular, and Marginal Zone B cells. Briefly, B cells were incubated with a mix of antibodies for 30 minutes, washed, and fixed with 1% paraformaldehyde. For B1 cells, a peritoneal exudate was obtained, and cells were stained with anti-CD19 BV421, anti-CD21 PE, and anti-CD5 PerCPCy5.5 (all from Biolegend). After incubation, cells were washed and fixed with 1% paraformaldehyde. Data was acquired using a FACS LSRFortessa™ (Beckton Dickinson, Franklin Lakes, NJ, USA). Data analysis was performed using FlowJo v10.10 (Beckton Dickinson). Percentages for each subpopulation were determined, and the total number was calculated using the total cell counts obtained from either the spleens or the peritoneal exudates.

### 2.4 Histological sections

Secondary organs, including the spleen, mesenteric lymph nodes, PP, and small intestine were obtained and placed in a tissue preservation medium (Leica, Wetzlar, Germany). They were then frozen in liquid nitrogen and stored at -70°C until use. Histological sections of 5–6 μm were mounted on slides coated with poly-L-lysine, fixed in cold acetone for 15 min, and stored at -20°C after drying at room temperature.

### 2.5 *In situ* immunofluorescent staining

Histological sections were rehydrated with 0.2% BSA in 1X PBS, blocked with Power Block Universal Blocking Reagent (BioGenex, CA, USA), and incubated for 1 h with anti-IgD (Becton Dickinson, 553438), anti-CD138 (Becton Dickinson, 553712), and biotinylated anti-IgA antibodies (eBioscience, San Diego, CA, USA, 13–5994-84). Subsequently, three washes with 0.2% BSA were performed, and anti-rat Alexa Fluor 594 secondary antibodies (Life Technologies, Carlsband, CA, USA, A21209) were added to detect anti-IgD and anti-CD138 antibodies, followed by incubation for 1 h at room temperature. Streptavidin coupled with Alexa Fluor 488 (Invitrogen, Waltham, MA, USA, 511223) was incubated with biotinylated primary antibodies for 30 min at room temperature. After staining, glass slides with histological sections were washed three times with 1× PBS, covered with coverslips, and mounted with 90% glycerol to keep the histological sections hydrated. Images were acquired using an Olympus BX51 microscope equipped with an Olympus U-CMAD3 camera and Olympus RFL-T epifluorescence lamp (Olympus, Tokyo, Japan) with 10× lenses. The analysis was performed using Image-Pro-Plus 7.0 (Media Cybernetics, Rockville, MD, USA) and Fiji ImageJ v2.7.0 software (18).

### 2.6 Flow cytometry for TGFβR detection

The spleens were obtained from the mice and disaggregated to create suspensions in 1× PBS. Cells were adjusted to 1×10^6^ cells in 50 μL of 1× PBS and stained with anti-TGFβRI PE (R&D, Minneapolis, USA, FAB5871P) at a 1:300 dilution, anti-TGFβRII (Abcam, ab61213) at a 1:100 dilution, and anti-CD19 BV605 (BD Pharmingen, 563148) at a 1:300 dilution, and viability dye with fixation eFLUORTM 450 (eBioscience) was added. The mixtures were incubated for 30 min. Subsequently, a secondary antibody for anti-TGFβRII, anti-rabbit coupled to Alexa Fluor 488 (Invitrogen, A21206), was added, and the cells were fixed with 1% paraformaldehyde.

For intracellular detection, after fixation with 4% PFA for 10 min, the cells were washed with 1× PBS. Blocking with 10% goat serum was performed for 30 min, followed by washing with 1× PBS. The cells were then permeabilized with a BD Perm/Wash™ (Becton Dickinson) for 10 min, followed by a final wash. Lymphocytes were then incubated with anti-TGFβRI PE and anti-TGFβRII for 1 h at 37°C. Subsequently, cells were washed with PBS and incubated with secondary antibodies against rabbit Alexa Fluor 488 in the case of the cell suspension with anti-TGFβRII. After the designated time, the cells were wash with 1X PBS was performed.

### 2.7 B-cell enrichment

Spleens were obtained from *Lrba-/-* and wild-type (WT) mice, disaggregated, and resuspended in 1x PBS. Subsequently, the MojoSort™ Mouse CD19 Nanobeads kit (BioLegend, San Diego, CA, USA) was used to enrich B cells, following the manufacturer’s instructions.

### 2.8 B-cell stimulation with TGFβ1

Purified B cells were adjusted to 3x10^6^ cells, resuspended in PBS, and placed in 1.5-mL tubes. Subsequently, 10 ng/mL of recombinant TGFβ1 (BioLegend, 763102) was added, and the cells were incubated at 37°C for 5, 15, and 30 min. Unstimulated cells served as controls. After stimulation, the cells were processed for immunoblotting.

### 2.9 Protein extraction and immunoblot analysis

The cells were lysed with 100 μL of lysis buffer (Cell Signaling Technologies, Danvers, MA, USA), containing 20 mM Tris-HCl (pH 7.5), 150 mM NaCl, 1 mM Na2EDTA, 1 mM EGTA, 1% Triton, 2.5 mM sodium pyruvate, 1 mM β-glycerophosphate, 1 mM Na3VO4, and 1 μg/mL leupeptin). Protease inhibitors (Roche, Mannheim, Germany) were added and the mixture was incubated for 30 min. The mixture was then centrifuged at 14,000 rpm for 15 min at 4°C. The protein concentration was determined using a BCA Protein Assay Kit (Pierce, Rockford, IL, USA). Samples were heated at 95°C for 5 min in Laemmli buffer (containing 1.0M Tris base pH 6.8, 10% SDS, 20% glycerol, 5% β-mercaptoethanol, and 0.01% bromophenol blue) and separated using 10% SDS-PAGE.

The proteins were transferred onto nitrocellulose membranes and blocked with 5% milk in TBS-Tween for 1 h at room temperature. Subsequently, the membranes were washed thrice for 10 min each with TBS-Tween, after which primary antibodies were added according to the specific assay requirements.

### 2.10 Immunoblots

Lrba and TGFβRI subunits were detected either in the immunoprecipitates, or total protein extracts were determined through immunoblotting, following standard procedures. Briefly, 20 μg of protein extracts from B cells were loaded onto a gradient gel 8–15% polyacrylamide (Bio-Rad, Hercules, CA, USA). A 10% polyacrylamide gel was used to detect the phosphorylated SMADs. The proteins were transferred to a nitrocellulose membrane for 1.5h at 100 V in standard Tris-glycine buffer with 20% methanol. The membranes were incubated overnight at 4°C with agitation using anti-Lrba, anti-TGFβRI (GeneTex, Zeeland, MI, USA, GTX134290), anti-TGFβRII, anti-phospho-SMAD2 (Cell Signaling, 138D4), anti-total SMAD 2/3 (Abcam, ab202445), and anti-Actin (Santa Cruz Biotechnology, Dallas, TX, USA). Subsequently, the membrane was washed, and a secondary antibody coupled to HRP was added and incubated for 1 h with agitation at room temperature. Finally, chemiluminescence detection was performed using Super Signal West Femto Maximum Sensitivity substrate on a ChemiDoc XRS (Bio-Rad), and images were acquired with a ChemiDoc™ (Bio-Rad).

### 2.11 Immunofluorescence staining for confocal microscopy

The previously purified B cells were adjusted to 1x10^6^ cells in 50 μL of 1× PBS and fixed with 4% PFA for 15 min. They were then washed with 1× PBS, followed by a 30-min blocking step with 10% goat serum. After another wash with 1× PBS, the fixed cells were permeabilized with BD Perm/Wash™ (Becton Dickinson, 51–2091 KZ) for 10 min. Following a second wash, the lymphocytes were incubated with goat anti-LRBA (Santa Cruz Biotechnology, sc-164907), rabbit anti-TGFβRI, or rabbit anti-TGFβRII antibodies for 1 h at 37°C. Subsequently, the cells were washed with PBS and incubated with secondary antibodies: anti-goat-Alexa Fluor 488 (Invitrogen, A11055), anti-rabbit-Alexa Fluor 594 (Jackson ImmunoResearch, West Grove, PA, USA, St Louis, MO, USA, 711–585-152), and DAPI (Sigma-Aldrich, 10236276001) for 1 h. Finally, the cells were washed and adhered to glass coverslips treated with poly-L-lysine (Sigma-Aldrich, P8920) for 1 h at 37°C. The preparations were mounted on slides using the VECTASHIELD mounting medium (Vector Laboratories, Newark, CA, USA, H-1000).

### 2.12 Confocal microscopy acquisition

Images were captured using a TCS SP-8 microscope (Leica Microsystems) at 63× magnification. Images were acquired using Leica LAS X software (Leica Microsystems). Co-localization analysis of samples with dual staining was conducted using Mander’s correlation coefficient for at least 50 cells from three independent experiments. Data analysis and Mander’s correlations were calculated using Fiji Image J software v2.7.0.

### 2.13 Statistical analysis

Results are presented as means ± standard deviation. Unless otherwise specified, non-parametric tests were used for statistical analysis, with *p* values <0.05 considered significant.

## 3 Results

### 3.1 *Lrba-/-* mice show high levels of IgA in both the serum and feces

**Figure 1:**
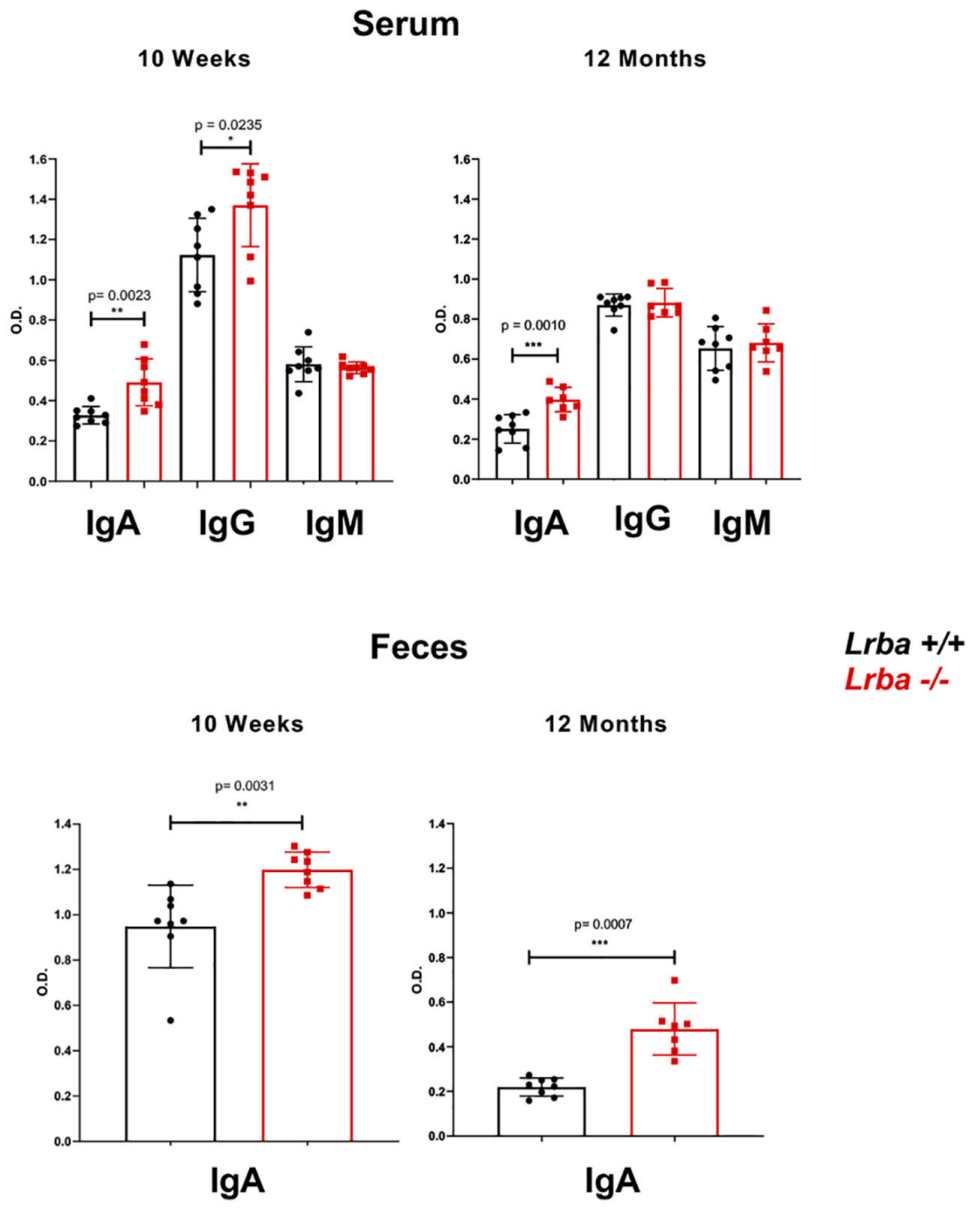
Immunoglobulin levels in serum and feces of Lrba-deficient mice aged 10 weeks and 12 months old. IgM, IgG, and IgA levels in serum samples (top) and for IgA stool samples (bottom) were determined using ELISA. Serum samples *Lrba +/+* n= 8 and *Lrba -/-* n= 8, stool samples *Lrba +/+* n= 8 and *Lrba* -*/-* n= 8. The Student´s t-test compared the means between the *Lrba+/+* and *Lrba-/-* mice, *p<0.05, **p<0.01, ***p<0.001. Similar data were previously published by Gamez-Diaz et al. (6) and by Burnett et al. (19).

The IgA levels in *Lrba-/-* were explored in serum and feces, we found elevated IgA levels in *Lrba-/-* mice. We observed higher levels of IgA in both the serum and feces of *Lrba-/-* mice at baseline, regardless of whether the mice were young (10 weeks) or old (12 months), compared with those in WT mice. We also observed higher levels of circulating IgG in young *Lrba*-deficient mice than that in WT mice (**Figure 1**). As Burnett et al. previously reported increased IgG2b levels, we determined this isotype in the sera from these mice; however, as shown in **Supplementary Figure 1**, similar IgG2b levels were observed in both mice. These results suggest potential alterations in B cell pathways that are essential for IgA production in *Lrba*-deficient mice. In addition, the mechanisms underlying the induction of the other isotypes were unaffected.

### 3.2 Altered B-cell subpopulations in *Lrba-/-* mice

**Figure 2:**
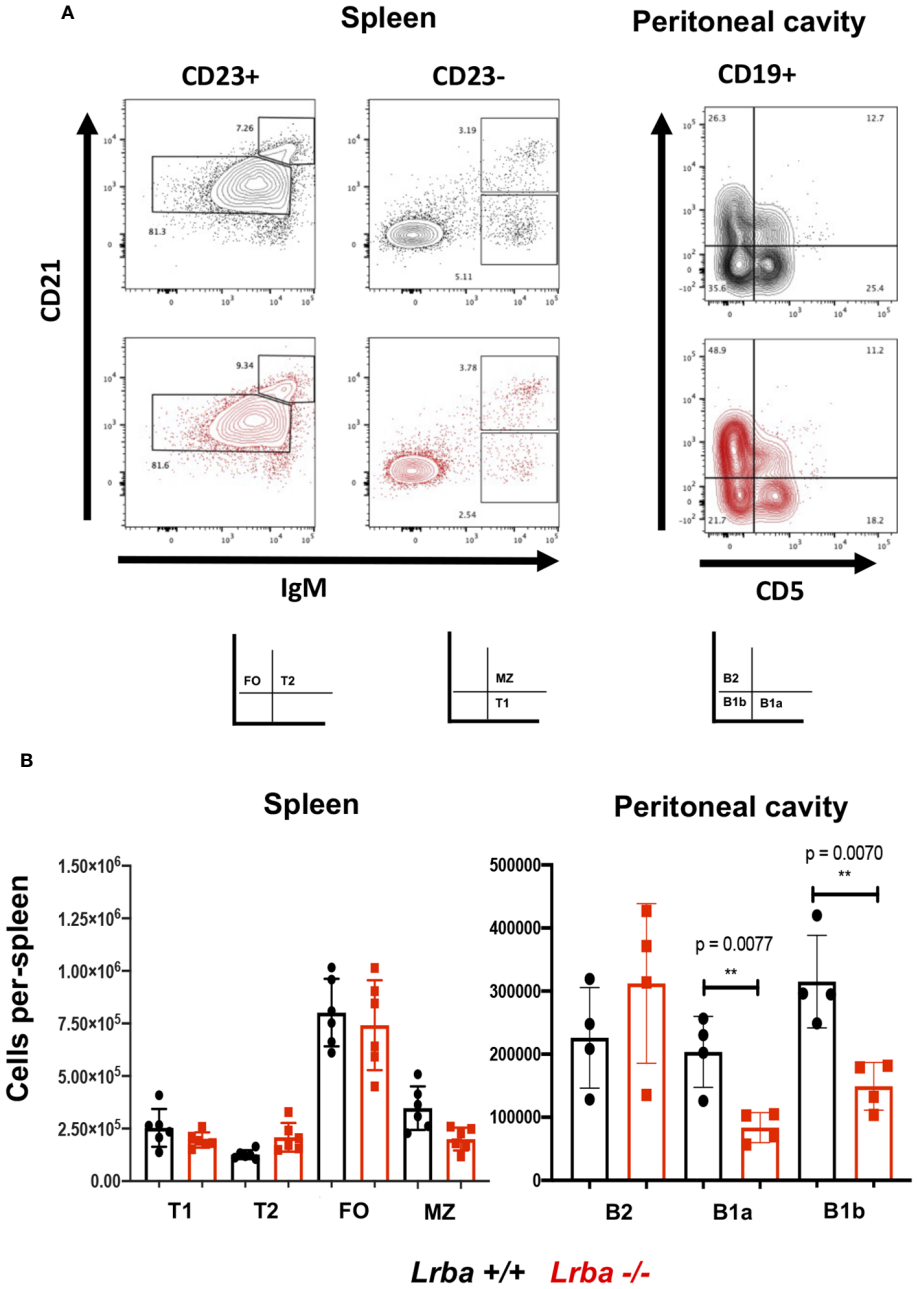
B-cell subpopulations in Lrba-deficient mice. **(A)** Representative image of B cell subpopulation analysis from spleen and peritoneal cavity. **(B)** Statistical analysis of B cell subpopulation in spleen from *Lrba+/+* n= 6 and *Lrba -/-* n= 6, and peritoneal B cells *Lrba +/+* n= 4 and *Lrba* -*/-* n= 4. The Student´s t-test compared the means between the *Lrba+/+* and *Lrba-/-* mice, **p<0.01. Similar data were previously published by Gamez-Diaz et al. (6) and by Burnett et al. (19).

After observing elevated IgA levels in *Lrba*-deficient mice, we assessed whether there was an alteration in the development of the spleen and B1 cell subpopulations in the peritoneal cavity. The spleen populations were not affected by the absence of Lrba. However, upon analyzing B1 lymphocyte subpopulations in the peritoneal cavity, we observed a decrease in both B1a and B1b subpopulations (**Figures 2A, B**).

### 3.3 The *Lrba-deficient* mice have more IgA+ B and plasma cells in secondary lymphoid organs and the small intestine

Normal B cell differentiation in the spleen and altered levels of B1a and B1b cells in the peritoneal cavity have led to an unclear source of IgA. To determine the lymphoid organs that predominantly produce IgA, we analyzed the mesenteric lymph nodes (MLN), PPs, and small intestine to detect the presence of IgA+ B cells and IgA+ plasma cells.

**Figure 3:**
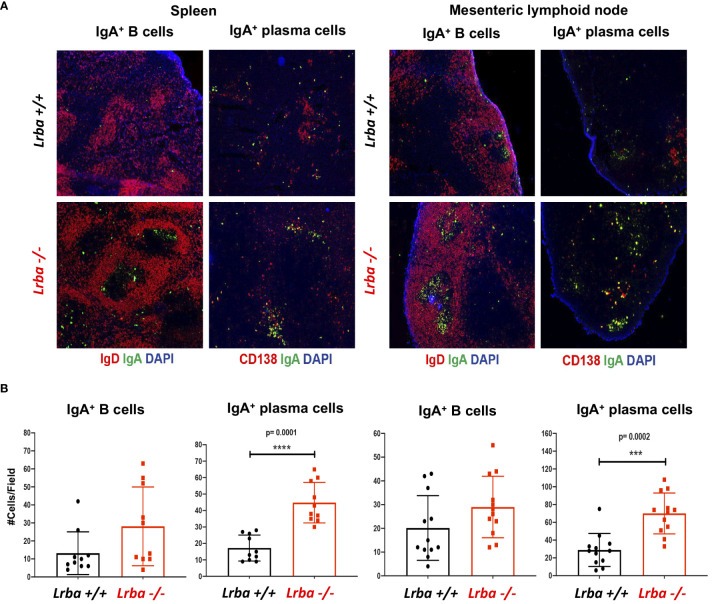
IgA+ B and plasma cells in histological sections from spleen and mesenteric lymph nodes from *Lrba-/-* and *Lrba+/+* mice. **(A)** Representative histological slices from spleen (left) and MLNs (right) stained with anti-IgA (green), anti-CD19 or CD138 (red) and DAPI. **(B)** Statistical analysis of IgA+ B and plasma cells counts per field. n=3 for both *Lrba+/+* and *Lrba-/-* mice. The Student´s t-test compared the means between the *Lrba+/+* and *Lrba-/-* mice, ***p<0.001, ****p<0.0001.

In the histological sections corresponding to the spleen and MLN, we observed a trend toward a higher number of IgA+ B cells and a more substantial presence of IgA+ plasma cells in both organs of *Lrba*-deficient mice (**Figures 3A, B**).

**Figure 4:**
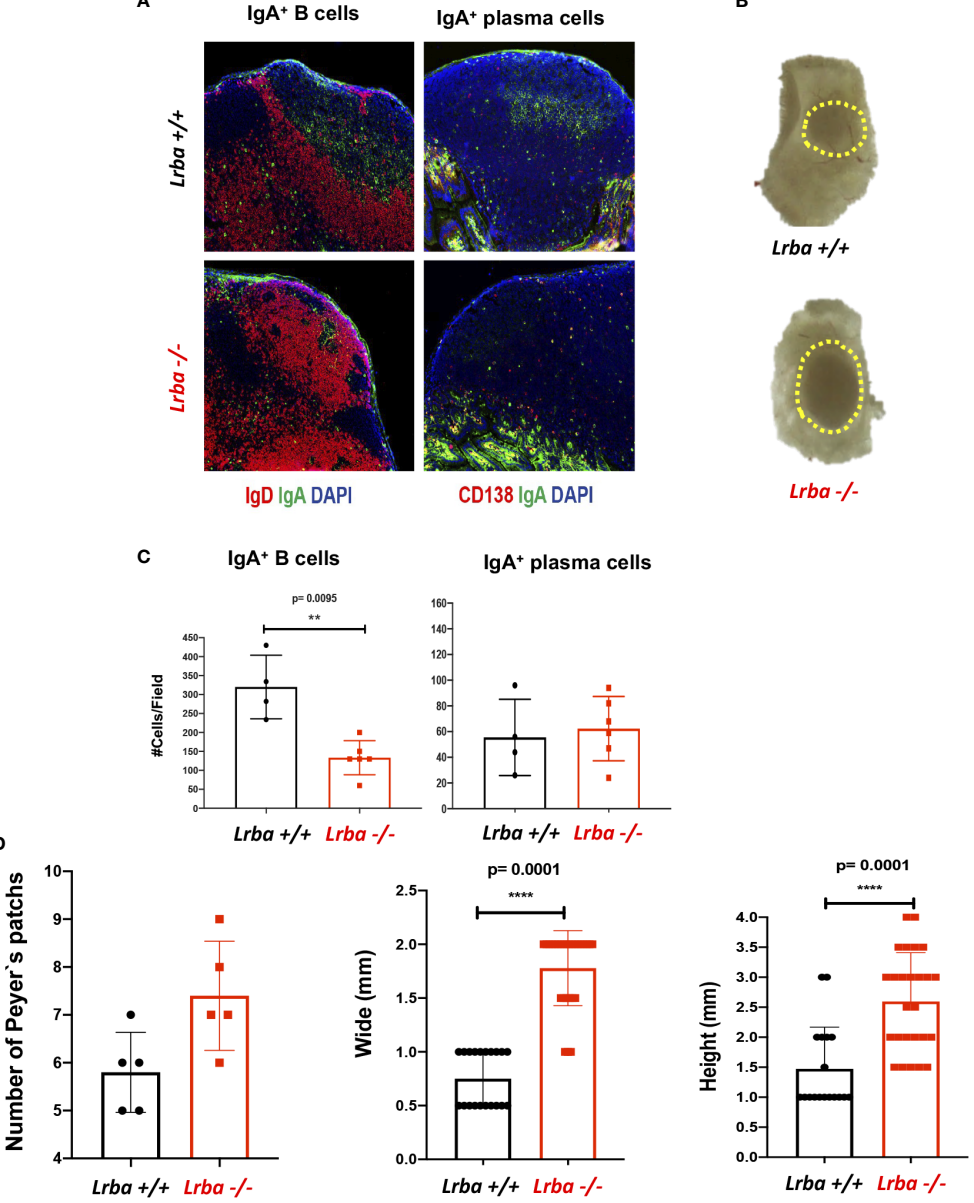
IgA+ B and plasma cells in histological sections from Peyer’s patches. **(A)** Representative histological slices from PPs stained with anti-IgA (green), anti-CD19 or anti-CD138 (red), and DAPI. **(B)** Representative image of a PP from *Lrba+/+* or *Lrba-/-* mice. **(C)** Statistical analysis of IgA+ B and plasma cells counts per field. **(D)** PPs counts (left) per intestine of *Lrba+/+* and *Lrba-/-* mice; PPs width (middle) and height (left) in *Lrba+/+* and *Lrba-/-* mice. n=3 for both *Lrba+/+* and *Lrba-/-* mice. The Student´s t-test compared the means between the *Lrba+/+* and *Lrba-/-* mice, **p<0.01, ****p<0.0001.

Smaller germinal centers were observed in the PP of *Lrba–* mice than that in WT, but with an evident size increase for these organs (**Figures 4A, B**). Additionally, a lower number of IgA+ B cells was counted in these organs, which contrasts with the results obtained in the spleen and MLN; however, upon determining the number of IgA+ plasma cells, we observed similar numbers in both mouse groups (**Figure 4C**). Analysis of the number of PPs in the small bowel of mice and measurement of the width and height of these organs confirmed a significantly higher number and size in *Lrba-/-* mice (**Figure 4D**). Considering the predominance of PPs in *Lrba-/-* mice, these lymphoid organs can be considered important sources of IgA.

**Figure 5:**
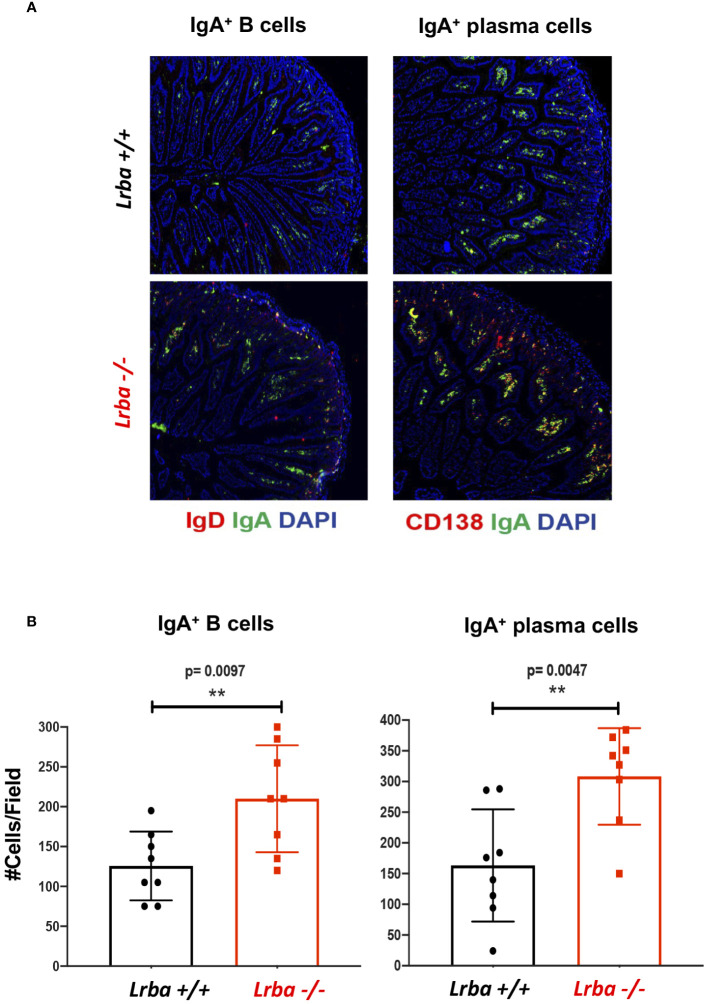
IgA+ B and plasma cells in histological sections from small bowel. **(A)** Representative histological slices from small bowel stained with anti-IgA (green), anti-CD19 or anti-CD138 (red), and DAPI. **(B)** Statistical analysis of IgA+ B and plasma cells counts per field. n=3 for both *Lrba+/+* and *Lrba-/-* mice. The Student´s t-test compared the means between the *Lrba+/+* and *Lrba-/-* mice, **p<0.01.

The small intestine was analyzed, as it is one of the sites where IgA+ plasma cells reside, produce, and secrete IgA. Notably, significantly higher number of IgA+ plasma cells were observed in *Lrba-/-* mice (**Figures 5A, B**).

Considering these results, the high levels of IgA in the serum and feces of *Lrba*-deficient mice could be explained by the increased number of IgA-producing plasma cells observed in all anatomical sites evaluated.

### 3.4 B cells from *Lrba-deficient* mice show reduced TGFβR expression

**Figure 6:**
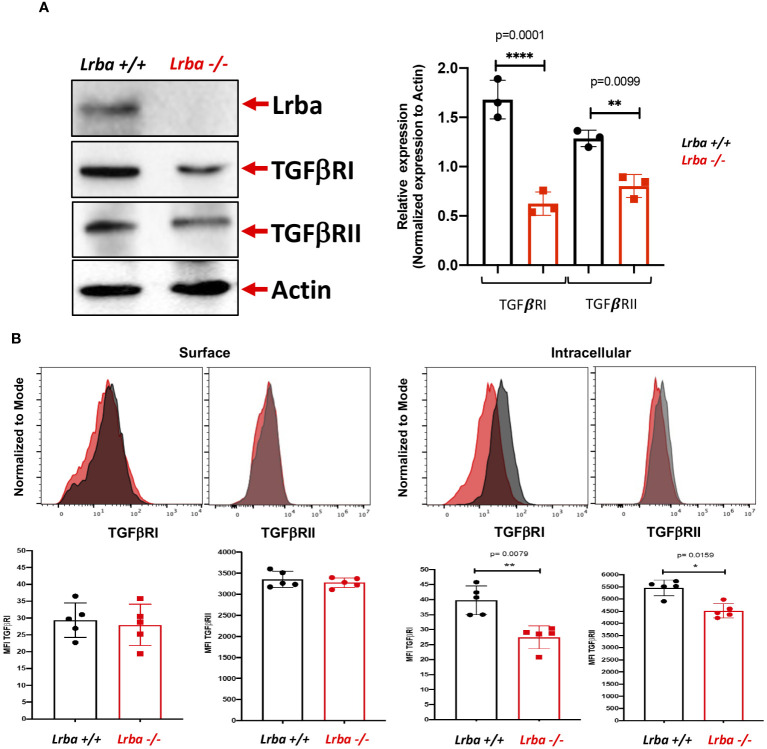
TGFβRI/TGFβRII expression by western blot and surface or intracellular detection by flow cytometry in B cells from *Lrba-/-* and *Lrba+/+* mice. **(A)** TGFβR (TGFβRI/II) detection using western blotting on purified B cells (left) and densitometric analysis of TGFβRI/II compared with b-actin as the loading control (right). **(B)** TGFβRI and II expression by surface (left) or intracellular (right) staining of B cells. Samples *Lrba+/+* n=6 and *Lrba-/-* n=6 mice. The Student´s t-test compared the means between the *Lrba+/+* and *Lrba-/-* mice, *p<0.05, **p<0.01, ****p<0.0001.

We analyzed aspects of the signaling pathway, including TGFβRI and TGFβRII expression in B cells. As shown in **Figure 6A**, *Lrba-/-* B cells have lower total TGFβRI and TGFβRII expression. As TGFβR is a membrane receptor that undergoes continuous recycling, we sought to determine if there was a difference in expression on the cell surface or intracellularly in B cells.

Surface and intracellular expression of TGFβRI is depicted in **Figure 6B**. There were no differences in the surface expression of this protein in B cells between the WT and *Lrba*-deficient mice. However, when analyzing the intracellular expression, we detected significantly lower expression of TGFβRI within the B cells of *Lrba*-deficient mice. Similarly, there were no differences in surface expression of TGFβRII, and a significant reduction in intracellular expression was also detected.

### 3.5 *Lrba*-deficient B cells showed increased SMAD2 phosphorylation in unstimulated conditions

After observing reduced intracellular expression of TGFβR in B cells from *Lrba*-deficient mice, SMAD2 phosphorylation was analyzed. Protein extracts were obtained from unstimulated purified splenic B cells or after stimulation with TGFβ1 at different time points (5, 15, and 30 min).

**Figure 7:**
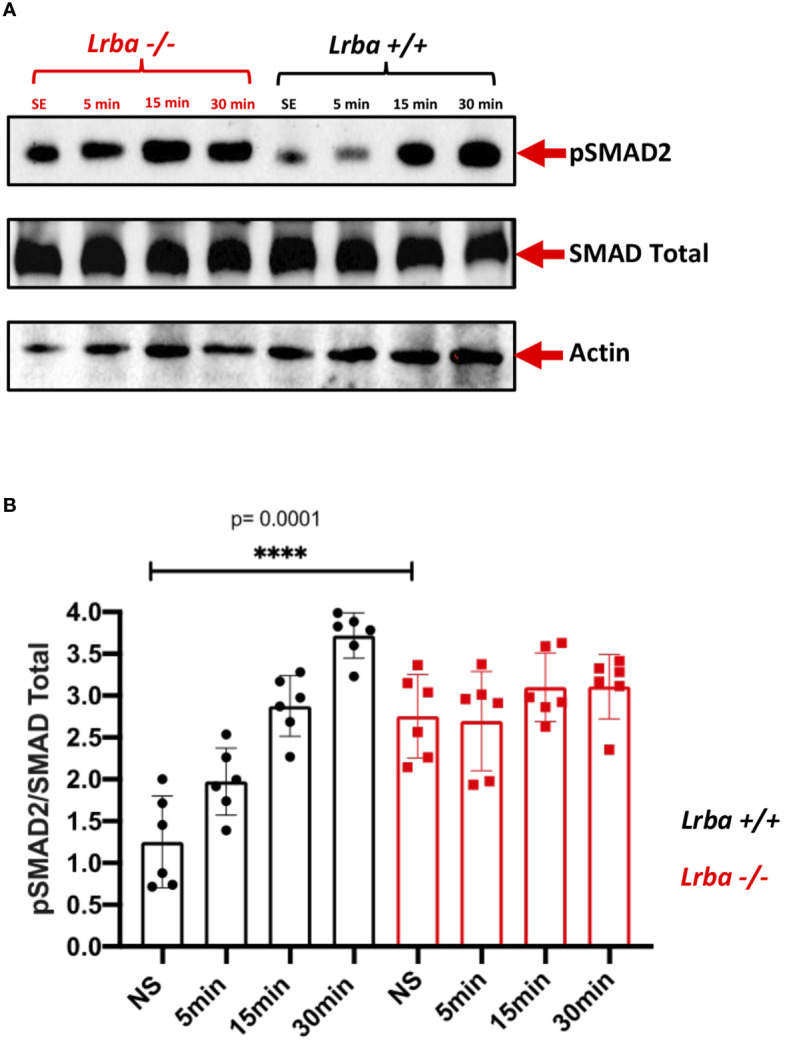
Phosphorylated SMAD2 expression in B cells from *Lrba*-deficient mice. **(A)** Phospho-SMAD2 detection using western blotting stimulated at different times point with TGFβ1 in purified spleenic B cells of *Lrba +/+* and *Lrba -/-.* **(B)** Densitometric analysis of phosphor-SMAD2 compared to total SMAD2 at different times of stimulation. Samples *Lrba+/+* n=6 and *Lrba-/-* n=6 mice. The Student’s t-test compared the means between the *Lrba+/+* and *Lrba-/-* mice, ****p<0.0001.

Notably, SMAD2 phosphorylation was significantly increased in unstimulated *Lrba-/-* B cells (**Figure 7**), whereas stimulation with the recombinant cytokine slightly induced SMAD2 phosphorylation, which remained similar to the basal levels throughout the entire kinetics (**Figures 7A, B**). This result contrasts with B cells from *Lrba+/+* mice, where initial phosphorylation was low, and it gradually increased with TGFβ1 stimulation. These data suggest that Lrba participates in controlling the activation of TGFβR.

### 3.6 Lrba co-localizes with both TGFβRI and TGFβRII in B cells

**Figure 8:**
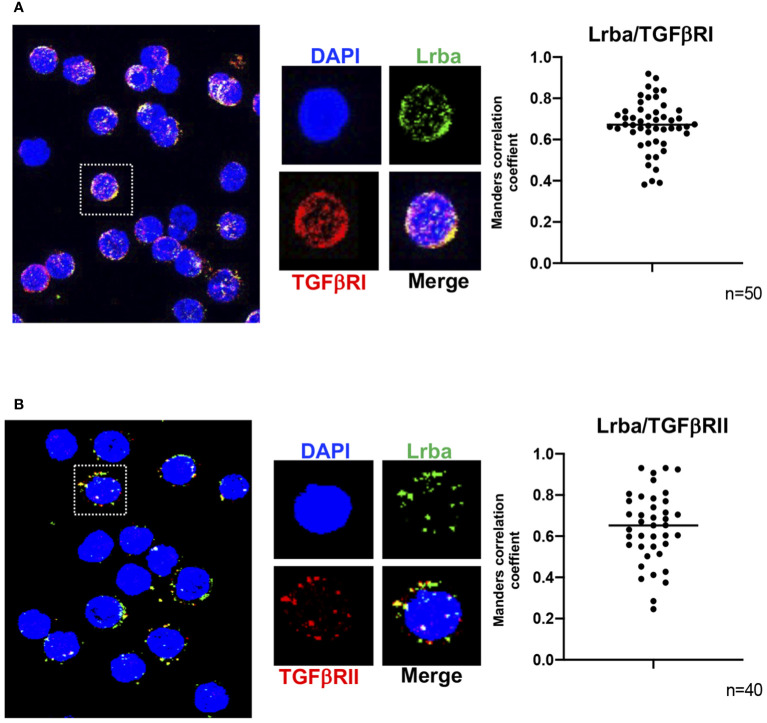
Colocalization of Lrba with TGFβR in B cells. **(A)** Representative image of TGFβRI/Lrba colocalization (left) and analysis of Mander’s correlation (right). **(B)** Representative image of TGFβRII/Lrba colocalization (right) and analysis of Manders correlation (right). n=3, the Student´s t-test compared the means between the *Lrba+/+* and *Lrba-/-* mice.

We analyzed whether TGFβR colocalizes with Lrba. **Figures 8A** and **B** show that Lrba co-localizes with both TGFβRI and II, respectively, with an approximate Mander’s correlation coefficient of around 0.7 (**Figure 8**). Co-immunoprecipitation experiments were also performed; however, the Lrba antibody did not sufficiently immunoprecipitate this protein, and only a weak signal was observed in both Lrba and TGFβRII immunoprecipitates (**Supplementary Figure 2**). Collectively, these results suggest that Lrba is involved in regulating TGFβR activation.

## 4 Discussion

LRBA has emerged as an important protein in immune processes, murine models can currently be used to study cellular functions involving Lrba. Here, we focused on elucidating the possible mechanism underlying the high levels of IgA in *Lrba-/-* mice.

We determined the levels of IgA in the serum and feces of young (10 weeks old) and old (12 months old) mice. Notably, the mice were not immunized. The data presented here reproduces previously published information on increased IgA levels. In contrast to previously published data (6), Significantly higher levels of IgG were detected in the sera of young mice in this study. Burnett et al. reported an increase in the IgG2b subclass in mice with a homozygous small deletion in the *Lrba* gene generated using CRISPR/Cas9 (19); however, we also determined the levels of IgG2b, which were not reproduced (**Supplementary Figure 1**). Additionally, we explored the *in vitro* induction of IgA+ and IgG2b+ B cells and their possible inhibition by TGFβRI SB505124. Upon stimulation, IgA+ B cells were properly induced, and the use of a TGFβR inhibitor (SB505124) blocked IgA+ B cell formation. This effect was not observed in the induction of IgG2b+ B cells (**Supplementary Figure 3**), suggesting that the increased levels of *Lrba-/-* mice are partly due to TGFβR activation; however, this switch to IgA requires other signals.

To determine whether the high levels of IgA production were due to a predominant B cell population, analysis of different B cell differentiation in the spleen and B1 subpopulations in the peritoneal cavity was performed. No alterations were detected in the proportion of B cells in the spleen; however, B1 cells were detected at reduced levels, which is consistent with the data reported by Gamez-Diaz et al. Explanations for the reduced B1 levels could result in poor survival, which has been reported for human LRBA-deficient B cells (2); B1 increased migration towards the intestine might be another possibility. B1 cells are precursors to a substantial percentage of intestinal IgA+ plasma cells in the lamina propria (20–22). Alterations in B1 cell maintenance, differentiation, or migration should be investigated in future studies.

To determine the source of the increased IgA levels, different secondary lymphoid organs, such as the spleen, MLNs, PPs, and small intestine, were examined. In all these organs, B cells can undergo isotype switching, not only to IgA, but also to other isotypes (23). Notably, all lymphoid organs explored, including the small intestine of *Lrba-/-* mice, showed a significantly higher number of IgA+ plasma and/or B cells than in WT mice. Importantly, although normal levels of IgA+ plasma cells were observed, significantly higher numbers and sizes were detected. These data suggest that all the explored organs contributed to the increased IgA production observed in both the feces and serum of *Lrba-/-* mice.

The increased sera IgA levels occur in non-immunized mice and the increased counts of IgA+ B and plasma cells suggest that the isotype switching to IgA occurs spontaneously in the *Lrba -/-* mice. The crucial role of TGFβ1 and TGFβR in T-dependent and independent class switch to IgA+ led us to propose TGFβR is an ideal candidate to be analyzed to explain the increased IgA production in *Lrba* deficiency.

To address this issue, TGFβR (I and II) expression was evaluated; the endocytosis of this receptor defines the intensity and duration of signaling upon contact with TGFβ1 (24). No differences were detected in the B-cell surface expression of the receptor. Importantly, a significant reduction in TGFβR I and II expression were found in permeabilized B cells from *Lrba -/-* mice. The altered expression of TGFβRI and II led us to evaluate the activation of SMAD2, the first signaling molecule activated after stimulation with the recombinant TGFβ1. High SMAD2 phosphorylation levels were observed in *Lrba-/-* B cells under non-stimulated conditions, confirming that Lrba participates in the TGFβR signaling pathway. This result was consistent with the hypothesis of spontaneous class switching to IgA. A previous report describes that high phosphorylation of SMAD2 leads to a preference for B cells to switch to IgA (25); however, that report described increased membrane expression of TGFβR in B cells that cannot form clathrin-coated vesicles. TGFβR signaling depends on endocytosis of the receptor upon interaction with TGFβ1 (26), and it has already been reported that it is internalized in endosomes coated with clathrin and caveolin (27). It is possible that in the absence of clathrin, the caveolin-dependent TGFβR endocytosis led to increased intensity of the signaling and promoting TGFβR expression. Additionally, another explanation for the normal expression of TGFβRI and II may be that TGFβR signaling also activates other pathways, such as PI3K-Akt-mTOR or MAPK (28, 29). In particular, Akt activation has been demonstrated to promote TGFβR membrane expression (30). However, we did not find any reports regarding PI3K-Akt-mTOR activation by TGFβR in B cells. Future studies should explore this issue.

SMAD2 phosphorylation was also examined in B1 and B cells derived from MLNs (**Supplementary Figure 4**). However, similar levels of pSMAD were detected between *Lrba-/-* and *Lrba+/+* B cells and the expression of CD38, an activation marker, was detected at similar levels in both murine strains, indicating that basal activation of the TGFβR pathway may be favored in splenic B cells.

Reduced intracellular TGFβRI/II and high SMAD2 phosphorylation in *Lrba-/-* splenic B cells confirmed alterations in this signaling pathway. Human and murine Lrba have high levels of similarity, therefore, these proteins may have similar functions. Additionally, CTLA4 membranal expression is decreased in *Lrba-/-* mice (6, 19). Human LRBA interacts with the Rab11 GTPase, a protein involved in recycling membrane receptors. TGFβR is recycled through the endocytic system, and also depends on Rab11-coated recycling endosomes for this (31). It is very likely that similar to CTLA4, Lrba is also required for effective TGFβR recycling, and its absence may favor TGFβRII degradation, which may explain the reduced intracellular expression observed in *Lrba-/-* B cells. LRBA role in CTLA-4 recycling was recently explored by Janman et al., who suggested, that the interaction between CTLA4 and Rab11 occurs only when LRBA is correctly expressed (32).

TGFβR also depends on Rab11 recycling endosomes, suggesting that Lrba can interact with TGFβR. Co-localization and co-immunoprecipitation experiments were performed to determine if Lrba can interact with TGFβRI and/or TGFβRII. The confocal microscopy experiments shown in **Figure 8** show a Mander’s correlation of Lrba colocalizing with both TGFβRI and II subunits with mean values of approximately 0.7, suggesting that Lrba may interact with both proteins and/or that Lrba is involved in the TGFβRI/II signaling pathway. As all commercially available antibodies are directed to human proteins, the negative control for Lrba staining is included in **Supplementary Figure 5**, indicating that the staining observed for Lrba is specific.

Finally, co-immunoprecipitation assays were performed, and the possible interaction between Lrba and TGFβR was determined, a faint Lrba band was detected in the immunoprecipitated TGFβRII and vice versa, suggesting the physical interaction between both proteins, *Lrba-/-* B cells were used as negative controls for this assay, observing a lack of Lrba bands in both Lrba and TGFβR immunoprecipitates (**Supplementary Figure 2**). It is possible that the high molecular weight of Lrba (320 KDa) may make it difficult to immunoprecipitate, in addition to the lack of antibodies directed to mice. The interaction between Lrba and TGFβR should be explored in future research to determine the exact Lrba domain(s) responsible for the interaction with TGFβRII and/or RI. Previously, Lo et al., demonstrated an interaction between LRBA and CTLA-4 via concanavalin A-like and PH-like domains (5). Given that TGFβR and CTLA-4 share no homology, the other domains of Lrba are likely responsible for such interactions.

Altogether, our results position Lrba as an essential molecule in controlling TGFβR signaling, influencing the differentiation mechanism of IgA+ B and plasma cells. Another important aspect is that while defects associated with TGFβR signaling have not been extensively described in humans, this work is an important starting point for investigating this signaling pathway in human diseases, such as cancer.

## Data availability statement

The original contributions presented in the study are included in the article/**Supplementary Material**. Further inquiries can be directed to the corresponding authors.

## Ethics statement

The animal study was approved by UPEAL-CINVESTAV-IPN, Protocol number: 145-15. The study was conducted in accordance with the local legislation and institutional requirements.

## Author contributions

JMFH: Writing – review & editing, Writing – original draft, Validation, Methodology, Investigation. FJHC: Methodology, Investigation, Writing – review & editing. DPP: Writing – review & editing, Investigation. HRR: Writing – review & editing, Methodology, Investigation. JCRA: Writing – review & editing. PLL: Writing – review & editing. MK: Writing – review & editing, Resources, Methodology. LSA: Writing – review & editing, Supervision, Resources, Conceptualization. GLH: Writing – review & editing, Supervision, Resources, Funding acquisition, Conceptualization.

## References

[1] Cullinane AR Schäffer AA Huizing M. The BEACH is hot: a LYST of emerging roles for BEACH-domain containing proteins in human disease. Traffic. (2013) 14:749–66. doi: 10.1111/tra.12069 PMC3761935 23521701

[2] Lopez-Herrera G Tampella G Pan-Hammarström Q Herholz P Trujillo-Vargas CM Phadwal K. Deleterious mutations in LRBA are associated with a syndrome of immune deficiency and autoimmunity. Am J Hum Genet. (2012) 90:986–1001. doi: 10.1016/j.ajhg.2012.04.015 22608502 PMC3370280

[3] Gámez-Díaz L August D Stepensky P Revel-Vilk S Seidel MG Noriko M. The extended phenotype of LPS-responsive beige-like anchor protein (LRBA) deficiency. J Allergy Clin Immunol. (2016) 137:223–30. doi: 10.1016/j.jaci.2015.09.025 26768763

[4] Wang JW Howson J Haller E Kerr WG. Identification of a novel lipopolysaccharide-inducible gene with key features of both A kinase anchor proteins and chs1/beige proteins. J Immunol. (2001) 166:4586–95. doi: 10.4049/jimmunol.166.7.4586 11254716

[5] Lo B Zhang K Lu W Zheng L Zhang Q Kanellopoulou C. AUTOIMMUNE DISEASE. Patients with LRBA deficiency show CTLA4 loss and immune dysregulation responsive to abatacept therapy. Science. (2015) 349:436–40. doi: 10.1126/science.aaa1663 26206937

[6] Gámez-Díaz L Neumann J Jäger F Proietti M Felber F Soulas-Sprauel P. Immunological phenotype of the murine Lrba knockout. Immunol Cell Biol. (2017) 95:789–802. doi: 10.1038/icb.2017.52 28652580

[7] Cerutti A. The regulation of IgA class switching. Nat Rev Immunol. (2008) 8:421–34. doi: 10.1038/nri2322 PMC3062538 18483500

[8] Islam KB Nilsson L Sideras P Hammarström L Smith CI. TGF-beta 1 induces germ-line transcripts of both IgA subclasses in human B lymphocytes. Int Immunol. (1991) 3:1099–106. doi: 10.1093/intimm/3.11.1099 1760405

[9] Peng SL. Signaling in B cells via Toll-like receptors. Curr Opin Immunol. (2005) 17:230–6. doi: 10.1016/j.coi.2005.03.003 15886111

[10] Mond JJ Vos Q Lees A Snapper CM. T cell independent antigens. Curr Opin Immunol. (1995) 7:349–54. doi: 10.1016/0952-7915(95)80109-X 7546399

[11] Litinskiy MB Nardelli B Hilbert DM He B Schaffer A Casali P. DCs induce CD40-independent immunoglobulin class switching through BLyS and APRIL. Nat Immunol. (2002) 3:822–9. doi: 10.1038/ni829 PMC4621779 12154359

[12] Dillon SR Gross JA Ansell SM Novak AJ. An APRIL to remember: novel TNF ligands as therapeutic targets. Nat Rev Drug Discov. (2006) 5:235–46. doi: 10.1038/nrd1982 16474316

[13] Kinoshita K Harigai M Fagarasan S Muramatsu M Honjo T. A hallmark of active class switch recombination: transcripts directed by I promoters on looped-out circular DNAs. Proc Natl Acad Sci USA. (2001) 98:12620–3. doi: 10.1073/pnas.221454398 PMC60103 11606740

[14] He B Qiao X Cerutti A. CpG DNA induces IgG class switch DNA recombination by activating human B cells through an innate pathway that requires TLR9 and cooperates with IL-10. J Immunol. (2004) 173:4479–91. doi: 10.4049/jimmunol.173.7.4479 15383579

[15] Fagarasan S Kawamoto S Kanagawa O Suzuki K. Adaptive immune regulation in the gut: T cell-dependent and T cell-independent IgA synthesis. Annu Rev Immunol. (2010) 28:243–73. doi: 10.1146/annurev-immunol-030409-101314 20192805

[16] Cazac BB Roes J. TGF-beta receptor controls B cell responsiveness and induction of IgA *in vivo* . Immunity. (2000) 13:443–51. doi: 10.1016/S1074-7613(00)00044-3 11070163

[17] Kurtenbach S Gießl A Strömberg S Kremers J Atorf J Rasche S. The BEACH protein LRBA promotes the localization of the heterotrimeric g-protein golf to olfactory cilia. Sci Rep. (2017) 7(1):8409. 28814779 10.1038/s41598-017-08543-4 PMC5559528

[18] Schindelin J Arganda-Carreras I Frise E Kaynig V Longair M Pietzsch T. Fiji: an open-source platform for biological-image analysis. Nat Methods. (2012) 9:676–82. doi: 10.1038/nmeth.2019 PMC3855844 22743772

[19] Burnett DL Parish IA Masle-Farquhar E Brink R Goodnow CC. Murine LRBA deficiency causes CTLA-4 deficiency in Tregs without progression to immune dysregulation. Immunol Cell Biol. (2017) 95:775–88. doi: 10.1038/icb.2017.50 PMC5636941 28611475

[20] Beagley KW Murray AM McGhee JR Eldridge JH. Peritoneal cavity CD5 (Bla) B cells: cytokine induced IgA secretion and homing to intestinal lamina propria in SCID mice. Immunol Cell Biol. (1995) 73:425–32. doi: 10.1038/icb.1995.66 8595920

[21] Kroese FG Ammerlaan WA Kantor AB. Evidence that intestinal IgA plasma cells in mu, kappa transgenic mice are derived from B-1 (Ly-1 B) cells. Int Immunol. (1993) 5:1317–27. doi: 10.1093/intimm/5.10.1317 7505612

[22] Fagarasan S Honjo T. T-Independent immune response: new aspects of B cell biology. Science. (2000) 290:89–92. doi: 10.1126/science.290.5489.89 11021805

[23] Macpherson AJ Slack E. The functional interactions of commensal bacteria with intestinal secretory IgA. Curr Opin Gastroenterol. (2007) 23:673–8. doi: 10.1097/MOG.0b013e3282f0d012 17906446

[24] Penheiter SG Mitchell H Garamszegi N Edens M Doré JJ Leof EB. Internalization-dependent and -independent requirements for transforming growth factor beta receptor signaling via the Smad pathway. Mol Cell Biol. (2002) 22:4750–9. doi: 10.1128/MCB.22.13.4750-4759.2002 PMC133902 12052882

[25] Wu S Majeed SR Evans TM Camus MD Wong NM Schollmeier Y. Clathrin light chains' role in selective endocytosis influences antibody isotype switching. Proc Natl Acad Sci USA. (2016) 113:9816–21. doi: 10.1073/pnas.1611189113 PMC5024586 27540116

[26] Hayes S Chawla A Corvera S. TGF beta receptor internalization into EEA1-enriched early endosomes: role in signaling to Smad2. J Cell Biol. (2002) 158:1239–49. doi: 10.1083/jcb.200204088 PMC2173232 12356868

[27] He K Yan X Li N Dang S Xu L Zhao B. Internalization of the TGF-β type I receptor into caveolin-1 and EEA1 double-positive early endosomes. Cell Res. (2015) 25:738–52. doi: 10.1038/cr.2015.60 PMC4456627 25998683

[28] Moustakas A Heldin CH. Non-smad TGF-beta signals. J Cell Sci. (2005) 118:3573–84. doi: 10.1242/jcs.02554 16105881

[29] Zhang YE. Non-smad signaling pathways of the TGF-β Family. Cold Spring Harb Perspect Biol. (2017) 9. doi: 10.1101/cshperspect.a022129 PMC5287080 27864313

[30] Duan D Derynck R. Transforming growth factor-β (TGF-β)-induced up-regulation of TGF-β receptors at the cell surface amplifies the TGF-β response. J Biol Chem. (2019) 294:8490–504. doi: 10.1074/jbc.RA118.005763 PMC6544840 30948511

[31] Yakymovych I Yakymovych M Heldin CH. Intracellular trafficking of transforming growth factor β receptors. Acta Biochim Biophys Sin (Shanghai). (2018) 50:3–11. doi: 10.1093/abbs/gmx119 29186283

[32] Janman D Hinze C Kennedy A Halliday N Waters E Williams C. Regulation of CTLA-4 recycling by LRBA and rab11. Immunology. (2021) 164:106–19. doi: 10.1111/imm.13343 PMC8358724 33960403

