# Supplementary material for: Lrba participates in the differentiation of IgA+ B lymphocytes through TGFβR signaling
Source: Front Immunol. 2024 Jun 21;15:1386260. doi: 10.3389/fimmu.2024.1386260 (PMC11224471; doi:10.3389/fimmu.2024.1386260)
Supplement: Supplementary file 1 [file DataSheet_1.docx]

Supplementary information

Supplementary methods

**Immunoprecipitation assays**

As described earlier, protein extracts from purified B cells were obtained and adjusted to a concentration of 500 μg in 500 μL. Once adjusted, a pre-clearing step was performed overnight at 4°C using Protein G Sepharose beads (Life Technologies, 101242). For immunoprecipitation, the pre-cleared protein extracts were incubated overnight with either rabbit IgG as an isotype control or with the following specific antibodies: anti-TGFβRII or anti-Lrba (Abcam, ab121601). After the initial incubation, the samples were further incubated overnight with Protein G Sepharose beads. Immunoprecipitates were washed three times with PBS and analyzed through immunoblotting.

**In vitro IgA induction**

3x10^5^ splenocytes were cultured in 200μl of supplemented RPMI and 100ng/ml of rmIL4. Cells were preincubated for 12 hours with or without 10μM SB505124 TGFβR1 inhibitor (Sigma-Aldrich). After incubation, stimuli were added without removing the inhibitor: 100ng/ml LPS O:111 (Sigma-Aldrich), 1mg/ml anti-IgM (Jackson Immunoresearch), 50μg/ml rCD40L (Peprotech) and 30ng/ml rTGFβ1, cells were incubated for 120 hours in 5% CO_2_ and 37^o^C. Cells were harvested and stained with anti-B220 APC. Intracellular staining was performed to detect IgA using the Perm/Wash buffer solution (Becton-Dickinson).

**pSMAD2 detection by flow cytometry**

5x10^5^ cells mixed with Fc block solution (Pharmingen) and stained with anti-CD19 BV421 Cells were washed and fixed for 10 minutes with Fix buffer I (Beckton Dickinson). Cells were washed and permeabilized for 30 minutes with Perm bufer III (Beckton-Dickinson), cells were washed twice with PBS 1% FCS and incubated overnight with anti-pSMAD2.


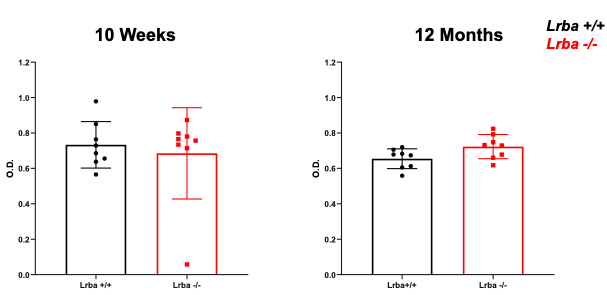


Supplementary Fig. 1. IgG2b detection in sera from *Lrba-/-* and *Lrba+/+* mice.


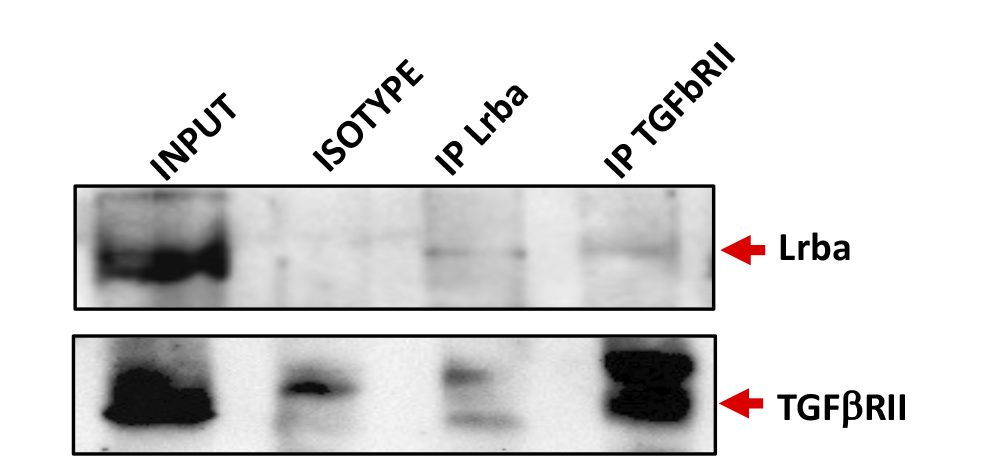


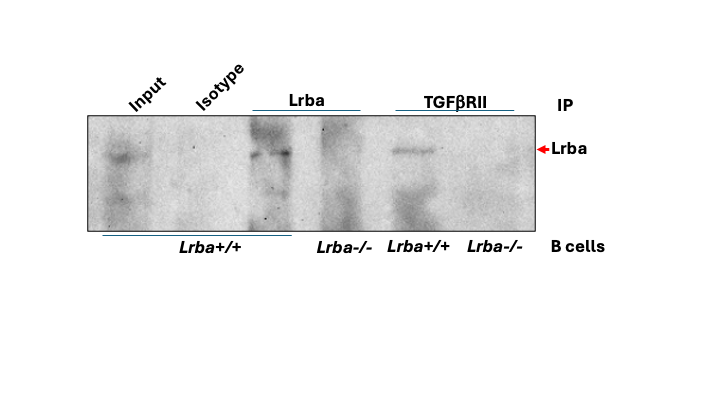


Supplementary Fig. 2. Lrba and TGFβRII coimmunoprecipitation. Lrba detection in both immunoprecipitates and TGFβRII detection in both immunoprecipitates from *Lrba+/+* B cells (top). Lrba detection in Lrba and TGFβRII immunoprecipitates from *Lrba+/+* and *Lrba-/-* B cells (bottom), showing that the signal observed for Lrba detection is specific.


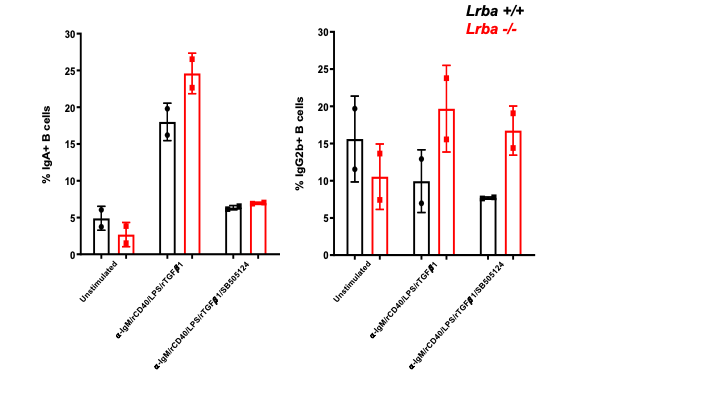
Supplementary figure 3. TGFβR1 inhibition reduces the IgA expression in *Lrba-/-* B cells. n=2.

Supplementary figure 4. Phosphorylated SMAD2 (left) and CD38 expression (right) in B1 (top) and B cells from mesenteric lymph nodes (bottom)


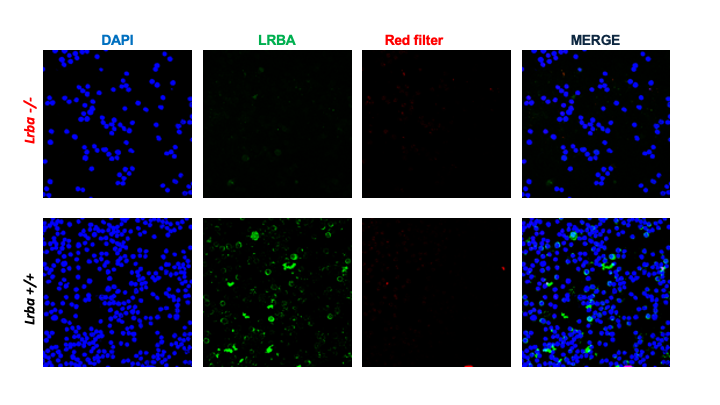


Supplementary figure 5. Negative control of Lrba staining in *Lrba-/-* B cells (top). Positive staining using *Lrba+/+* B cells (bottom).
